# Comparative Analysis of Insect Resistance in Transgenic *Populus × euramericana* cv. Neva Expressing Dual *Bt* Genes from Different Sources

**DOI:** 10.3390/plants15010051

**Published:** 2025-12-23

**Authors:** Jialu Li, Jiali Zhang, Hongrui Li, Chunyu Wang, Xue Yan, Yachao Ren, Jinmao Wang, Minsheng Yang

**Affiliations:** 1 Institute of Forest Biotechnology, Forestry College, Agricultural University of Hebei, Baoding 071000, China; lijialu1609@163.com (J.L.); 18833058627@163.com (J.Z.); lihongruiu1111@126.com (H.L.); chunyuwang2024@163.com (C.W.); 18903541094@163.com (X.Y.); rycyx@126.com (Y.R.); 2 Hebei Key Laboratory for Tree Genetic Resources and Forest Protection, Baoding 071000, China

**Keywords:** transgenic *Bt* poplar 107, insect resistance, exogenous gene expression, *Hyphantria cunea*, *Plagiodera versicolora*, *Anoplophora glabripennis*, transcriptome analysis

## Abstract

This study systematically evaluated insect resistance in transgenic poplar lines carrying three distinct *Bacillus thuringiensis* (*Bt*) gene vector architectures: a single-gene pb vector (*Cry1Ac*), a reverse-oriented double-gene n19 vector (*Cry1Ac*-*Cry3A*), and a forward-oriented double-gene n5 vector (*Cry3A*-*Cry1Ac*). The transgenic lines were accordingly designated as pb8/pb9, n19a/n19b, and DB7/DB16, respectively. Molecular analyses confirmed stable *Bt* gene integration, with the expression of *Cry3A* being consistently higher than that of *Cry1Ac* expression. Bioassays showed that dual-gene lines conferred broader insect resistance to pests than that of single-gene lines against both lepidopteran (*Hyphantria cunea*) and coleopteran (*Plagiodera versicolora*, *Anoplophora glabripennis*) pests. In contrast, the single-gene line pb9 exhibited specialized, high efficacy against *H. cunea*, achieving 100% mortality. Transcriptomic analysis of *P. versicolora* larvae fed the double-gene high-resistance n19a line and low-resistance DB16 line revealed multi-level molecular responses to Bt stress, including up-regulation of toxin-activating proteases, altered receptor expression, and suppression of growth-related genes. These changes were associated with significant developmental delay (8.33–20.83% reduction in the molting index). Our findings characterize the insect resistance and molecular profiles of the six transgenic poplar lines, as follows: multi-gene lines (n19a/n19b and DB7/DB16) confer broad-spectrum pest resistance, whereas single-gene lines (pb8/pb9) exhibit targeted efficacy. These results support the utility of these lines for pest-specific poplar breeding programs.

## 1. Introduction

The insecticidal crystal protein genes derived from *Bt* constitute a major genetic resource in agricultural and forestry breeding [1]. For example, Cry1Ac and Cry3A—two well-characterized Bt insecticidal proteins—target Lepidoptera and Coleoptera pests, respectively. As a cornerstone of plant biotechnology, Bt toxins were identified early and successfully deployed in the development of insect-resistant plants—a pivotal advancement that laid the foundation for modern genetic approaches to crop protection [2]. Since their discovery, they have been widely adopted in agricultural and forestry genetic engineering breeding. Through genetic engineering technologies, *Bt* genes have been widely incorporated into high-performing crops and fast-growing trees, such as cotton [3], maize [4], and poplar [5], creating novel germplasm that combines productivity with insect resistance. 

It should be noted that Bt toxins are not exclusively a product of transgenic technology; long before the commercialization of transgenic insect-resistant plants, Bt-based microbial formulations had already been widely used for field pest control [6]. This longstanding history of practical use provides not only empirical evidence of environmental safety but also a conceptual foundation for the subsequent development of transgenic insect-resistant plants [7]. Among *Bt* toxins, the Cry family has been studied most extensively, exhibiting insecticidal activity against a broad range of pests, including Lepidoptera, Coleoptera, Diptera, Hemiptera, and Hymenoptera, as well as certain nematodes and mollusks. The insecticidal mechanism of Cry proteins (e.g., *Cry1Ac* and *Cry3A*) involves their binding to specific receptors—such as cadherin (CAD), aminopeptidase N (APN), and alkaline phosphatase (ALP) [8,9,10,11]. This binding induces pore formation in the epithelial cells, disrupting osmotic balance and ultimately leading to insect death [12,13]. The insecticidal specificity of Cry proteins is determined by structural variations in their domains II and III, which arise from the long-term coevolution between *B. thuringiensis* and insect hosts. Differences in the molecular structure of midgut receptors define the recognition specificity of Cry toxins, resulting in a highly targeted insecticidal spectrum [14,15,16,17,18].

Stable integration and efficient expression of exogenous genes in the plant genome are essential for the successful application of transgenic trees. Studies confirm that inserted genes can indeed be maintained stably over time. For example, in seven transgenic apple lines carrying the *GFP* gene, all retained kanamycin resistance and showed stable *GFP* expression even after nine years of continuous subculture [19]. Similarly, analysis of two-year-old transgenic poplar 107 (a hybrid clone of *Populus × euramericana* ‘Neva’) carrying *Bt Cry1Ac*/*Cry3A* genes in an experimental plantation revealed variations in transcript abundance and toxin protein levels across six tested lines [20]. However, stable gene presence does not guarantee effective expression. Expression is regulated by multiple factors: the arrangement and orientation of genes in multi-gene expression vectors significantly affect expression levels [21]; high copy numbers often induce gene silencing, whereas one–two copies generally support favorable expression [22,23]; environmental stresses—such as cold-moist and dry-heat conditions—can modulate exogenous gene expression [24]; insertion-site preference exists, with integration favoring transcriptionally active regions, whereas insertion into silenced or repressed regions can lead to partial or full suppression of gene expression [20,25].

*P. × euramericana* ‘Neva’ (cultivar designation ‘74/76’, commonly called Poplar 107) is a hybrid clone of *P. nigra* L. (European black poplar) and *P. deltoides* Bartr. (eastern cottonwood) within the genus *Populus* L., family Salicaceae. As a predominant cultivated variety in Northern China, it grows rapidly, possesses excellent wood properties, and is easily propagated vegetatively, making it well suited for panel products and pulp materials [26]. However, large-scale monoculture plantations have exacerbated pest problems. Outbreaks of insects such as *H. cunea*, *A. glabripennis*, and *P. versicolora* can cause severe tree damage and even trigger widespread stand mortality, threatening both the ecological and economic value of poplar plantations [27]. Because of the narrow insecticidal spectrum and selective activity of individual *Bt* genes, the strategy for engineering *Bt*-mediated insect resistance has shifted from single-gene transformation to multi-gene co-transformation in response to increasing pest pressures. However, problems such as transgene loss, silencing, and variable expression often limit the efficiency of multi-gene breeding.

This study systematically evaluated six established transgenic poplar 107 lines carrying different *Bt* gene configurations: two single-gene lines (pb8 and pb9) [28] expressing *Cry1Ac*, two lines with *Cry1Ac* and *Cry3A* genes arranged in opposite transcriptional orientations (n19a and n19b) [29], and two lines with the two genes oriented in the same direction (DB7 and DB16) [30]. Using wild-type poplar 107 as control, we assessed the stability of exogenous gene expression and the resulting resistance across these lines. This work aims to clarify how different *Bt* gene configurations shape pest resistance in poplar 107, offering targeted theoretical support for the breeding of multi-gene insect-resistant poplar. In addition, we performed transcriptome analysis on *P. versicolora* larvae fed leaves from a high-resistance line (n19a) and a low-resistance line (DB16) to elucidate molecular response patterns and explore interaction mechanisms between Bt toxins and insects.

## 2. Results

### 2.1. PCR Detection of Exogenous Genes in Transgenic Lines

**Figure 1:**
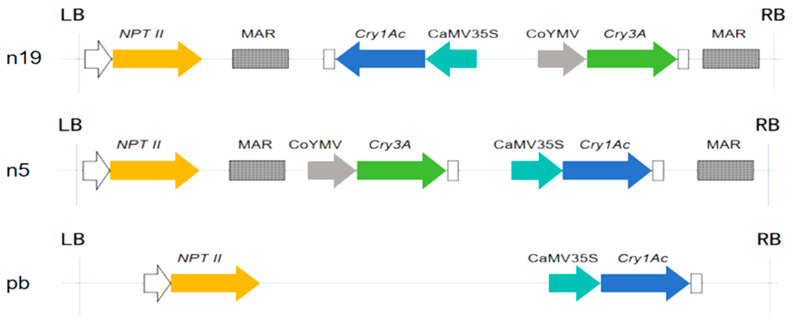
Schematic diagrams of plant expression vector constructs (n19, n5, and pb). All symbols are defined as follows: LB/RB: left/right border of T-DNA (mediates T-DNA integration into plant genome); *NPTII*: neomycin phosphotransferase II gene (selectable marker for kanamycin resistance); MAR: matrix attachment region (enhances transgene expression and reduces position effect); CaMV35S: cauliflower mosaic virus 35S promoter (constitutive strong promoter); CoYMV: commelina yellow mottle virus promoter (vascular tissue-specific promoter); *Cry1Ac*/*Cry3A*: Bt insecticidal protein genes (targeting lepidopteran/coleopteran pests).

A schematic of the T-DNA regions for each vector is provided to illustrate the genetic constructs used in this study (Figure 1). To confirm the integration of exogenous genes, molecular characterization was then performed using PCR on genomic DNA from six transgenic lines and wild-type (WT) controls. All transgenic lines yielded target bands of 665 bp (corresponding to the neomycin phosphotransferase gene *nptII*) and 602 bp (corresponding to the *Cry1Ac* gene). Lines n19a, n19b, DB7, and DB16 also showed a 667 bp band (corresponding to the *Cry3A* gene), confirming the stable presence of both the selectable marker and the *Bt* genes in these plants (Supplementary Figure S1).

### 2.2. Analysis and Validation of Exogenous Gene Insertion Sites in Transgenic Lines

To precisely characterize the genomic integration patterns of the T-DNA constructs, whole-genome sequencing (WGS) was performed on the six transgenic lines. After sequencing, approximately 129 Gb of high-quality data were obtained, with Q30 scores exceeding 91% for all lines (Supplementary Table S1). Alignment analysis identified nine insertion sites across the six lines, located primarily on chr1, chr2, chr5, chr10, chr12, and chr17 (Table 1 and Table S2). Analysis of genes near the insertion sites showed that, except for n19b, which was inserted within the coding region of gene *H0E87_011016*, all other insertions occurred in intergenic regions. Notably, transgenic lines generated using the n5 vector (DB7 and DB16) exhibited multiple insertion sites: DB16 contained insertions on chr1, chr12, and chr17, while DB7 had two insertion sites on chr10.

**Figure 2:**
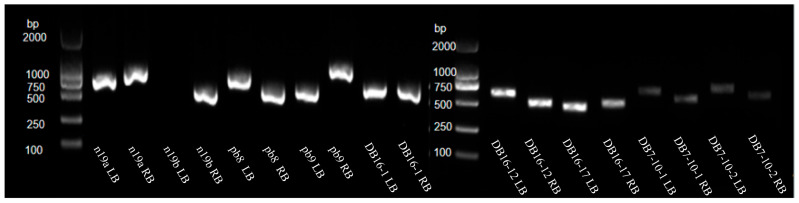
Validation of exogenous gene insertion sites in the transgenic line by PCR analysis.

To validate the insertion sites and orientations identified by WGS, further PCR-based confirmation was carried out for each line. Specific primers were designed based on the flanking genomic sequences (Supplementary Table S3) and paired with vector-specific primers to verify T-DNA insertion locations and directions (primer combinations are listed in Supplementary Table S4). Using these primer sets, genomic DNA from each line was amplified (Figure 2). All insertion sites were successfully validated except for the left border of n19b, which did not yield a detectable product. Some lines produced amplification fragments smaller than expected, which may reflect deletions in the T-DNA region or adjacent genomic sequences.

### 2.3. Analysis of Exogenous Gene Expression in Transgenic Lines

Following successful RNA extraction (Supplementary Figure S2) and reverse transcription, qRT-PCR generated specific amplification curves with distinct Ct values for each transgenic line. Standard curves were generated using serially diluted cDNA standards. The standard curve equation for the *Cry1Ac* gene was y = −3.493log(x) + 57.29 (r = 1.000) (Supplementary Figure S3), and for the *Cry3A* gene was y = −3.433log(x) + 48.77 (r = 0.997), both demonstrating excellent linearity (Supplementary Figure S4).

**Figure 3:**
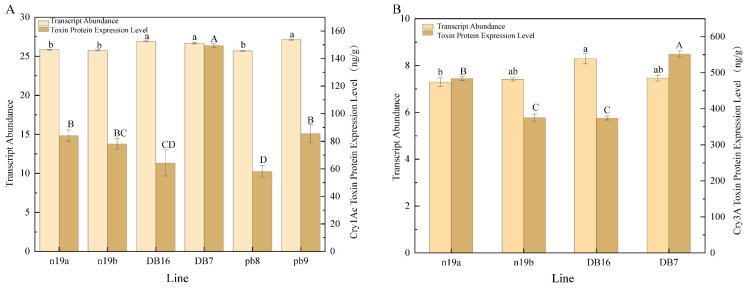
Transcriptional abundance of exogenous *Cry* genes and Bt toxin accumulation in transgenic lines. (**A**) Transcriptional level of the *Cry1Ac* gene and content of the corresponding Cry1Ac toxin in different transgenic lines. (**B**) Transcriptional level of the *Cry3A* gene and content of the corresponding Cry3A toxin in different transgenic lines. All data are presented as mean ± SD (n = 3). For both (**A**,**B**), lowercase letters above bars indicate significant differences in transcriptional abundance among transgenic lines, while capital letters above bars indicate significant differences in toxin content among transgenic lines, as determined by multiple comparisons (*p* < 0.05). Source data are provided as a Supplementary Data File.

Transcript abundance of *Bt* genes varied across transgenic lines (Figure 3). The transcript levels of the *Cry1Ac* gene ranged from 25.69 ± 0.04 to 27.11 ± 0.09, while those of *Cry3A* ranged from 7.29 ± 0.19 to 8.29 ± 0.22. Significant differences in transcript abundance were observed between several lines. The highest *Cry1Ac* level was detected in line pb9, and the lowest in pb8. For the *Cry3A*, the highest transcript abundance occurred in DB16, and the lowest in n19a.

Comparative analysis of expression levels revealed distinct patterns among lines. For *Cry1Ac* gene expression, DB7/DB16 (n5-derived lines) showed the highest transcript abundance, followed by pb8/pb9 (pb-derived lines), whereas n19a/n19b (n19-derived lines) were relatively lower.

ELISA analysis revealed that Cry1Ac toxin protein expression levels ranged from 58.00 ± 4.23 to 149.42 ± 1.41 ng·g^−1^, while Cry3A toxin protein expression levels ranged from 374.49 ± 5.58 to 552.41 ± 8.45 ng·g^−1^ among the transgenic lines. At the protein level, however, pb vector lines accumulated the most Cry1Ac toxin content, followed by n19 vector lines, with n5 vector lines exhibiting the lowest accumulation. For *Cry3A*, n5 vector lines performed best in both transcript abundance and toxin protein accumulation, followed by n19 vector lines. Substantial variation in toxin protein expression was observed across different transgenic lines. The highest *Cry1Ac* expression was detected in DB7, and the lowest in pb8. For *Cry3A*, the highest protein level was found in DB7, and the lowest in DB16.

### 2.4. Insect Resistance Assessment of Transgenic Lines

#### 2.4.1. Analysis of Resistance to *H. cunea* in Transgenic Lines

##### Analysis of Resistance Against 1st-Instar *H. cunea* Larvae

**Figure 4:**
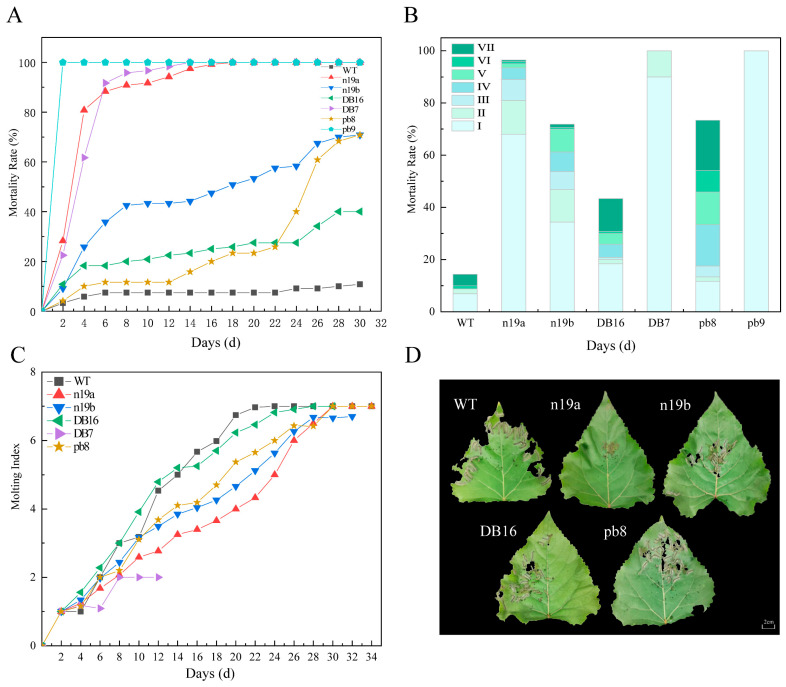
Insect resistance of transgenic lines against 1st-instar larvae of *H. cunea*. (**A**) Mortality dynamics of 1st-instar *H. cunea* larvae with feeding days. (**B**) Mortality of *H. cunea* larvae at each instar stage under transgenic lines. Roman numerals I–VII denote the larval instar stage. (**C**) Ecdysis index changes of 1st-instar *H. cunea* larvae with feeding days (all larvae in the PB9 group died within two days, and no molting was observed). (**D**) Comparison of larval leaf feeding area across different lines after six hours on day 14 (larvae in pb9 and DB7 groups were all dead and therefore not labeled in the figure). The scale bar is approximate; the figure is presented as a schematic representation.

Significant differences in lethal efficacy against 1st-instar *H. cunea* larvae were observed among the transgenic lines (Figure 4A). Lines pb9, DB7, and n19a exhibited rapid toxic effects, with mortality rates exceeding 80% by day 6, and reaching 100% within 2, 14, and 18 days, respectively. In contrast, lines n19b, pb8, and DB16 showed slower progression, reaching respective mortality rates of 68.33%, 67.33%, and 40% after 32 days of feeding. Specifically, pb8 initially caused gradual lethality but showed a sharp rise after day 22, with mortality rising by 35% over 4 days. DB16 also accelerated after day 24, rising 12.5% in 4 days, while n19b rapidly reached 33.33% mortality within the first 8 days, then plateaued. When grouped by vector construct, lines carrying the pb vector (pb8, pb9) demonstrated the strongest lethal effects on 1st-instar larvae, followed by n19 vector lines (n19a, n19b), whereas n5 vector lines (DB7, DB16) performed relatively poorly.

Developmental inhibition of 1st-instar larvae also varied among the transgenic lines (Figure 4B). Among larvae fed leaves of pb9, DB7, and n19a lines, over 68% died during the 1st instar. This proportion exceeded 40% for the n19b and DB16 lines. In contrast, only 16% of larvae feeding on pb8 died at the 1st instar, while a higher proportion (26%) died at the 7th instar. The pupation rates for n19b, pb8, and DB16 lines were relatively high, at 28.13 ± 3.56%, 56.67 ± 5.24%, and 85.63 ± 4.45%, respectively.

Analysis of the molting index (Figure 4C) revealed that larvae feeding on leaves from transgenic lines exhibited varying degrees of developmental inhibition compared with the WT controls. Due to the complete mortality of larvae feeding on the DB7 line after 12 days, their molting index recording was terminated. Larvae consuming n19b and pb8 lines exhibited molting indices lower than WT, demonstrating significant inhibitory effects from the early experimental stages. In contrast, larvae fed the DB16 line showed no apparent inhibition initially, with molting indices even exceeding WT, before gradually displaying inhibitory effects after day 16, when the index dropped below WT levels. Furthermore, all transgenic lines prolonged the time required for larval pupation. Compared with WT (22 days), the pupation time for n19b, pb8, and DB16 lines was extended by 12, 8, and 6 days, respectively, while pb9 and DB7 lines completely failed to pupate. Regarding molting index inhibition, lines carrying the pb vector consistently maintained the lowest levels, indicating optimal developmental inhibition efficacy.

On day 14 of the experiment, larval feeding area was recorded 6 h after supplying leaves from each line (Figure 4D). The results showed significant differences in the feeding area of 1st-instar *H. cunea* larvae on different transgenic lines. WT leaves showed the most severe feeding damage with extensive tissue loss, whereas n19a leaves remained relatively intact. The other lines exhibited varying degrees of leaf consumption.

##### Analysis of Resistance Against 3rd-Instar *H. cunea* Larvae

**Figure 5:**
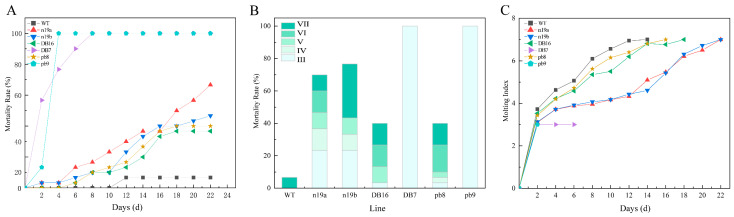
Insect resistance of transgenic lines on 3rd-instar *H. cunea* larvae. (**A**) Mortality dynamics of 3rd-instar *H. cunea* larvae with feeding days. (**B**) Mortality of *H. cunea* larvae at each instar stage under transgenic lines. Roman numerals III–VII denote the larval instar stage. (**C**) Ecdysis index changes of 3rd-instar *H. cunea* larvae with feeding days.

Significant differences in lethal efficacy against 3rd-instar *H. cunea* larvae were observed among transgenic lines, with some lines showing reduced toxicity compared with their effects on 1st-instar larvae (Figure 5A). Notably, lines pb9 and DB7 still exhibited rapid toxic action, achieving 100% mortality within 4 and 8 days, respectively. Compared with 1st-instar larvae, the time required for complete mortality differed for 3rd-instar larvae: extended from 2 to 4 days for pb9, but shortened from 14 to 8 days for DB7. The lethal efficacy of n19a increased gradually over time, but was significantly lower against 3rd-instar than 1st-instar larvae, showing a 33.33% reduction by day 22. Lines n19b, pb8, and DB16 exhibited relatively mild effects initially, followed by rapid increases from day 10, reaching mortality rates of 23.33~30% mortality within 6 days. Their final mortality rates against 3rd-instar larvae were 11.67%, 3.33%, and 28.33% lower than against 1st-instar larvae, respectively.

The developmental inhibition of 3rd-instar larvae also varied across transgenic lines (Figure 5B). All larvae feeding on pb9 and DB7 lines died during the 3rd instar. A high proportion (33.33%) of larvae fed n19a died at the 3rd instar, whereas n19b showed a high proportion (33.33%) at the 7th instar. No DB16 larvae died in the 4th instar, while over 41.67% of pb8 larvae died at the 6th instar. Pupation rates for n19a, n19b, DB16, and pb8 lines were 30 ± 1.41%, 23.33 ± 0.94%, 60 ± 1.41%, and 60 ± 0%, respectively. When grouped by vector construct, lines carrying the pb vector exhibited the strongest resistance against 3rd-instar larvae, followed by those with the n5 vector, while the n19 vector lines remained relatively weaker.

Molting index analysis (Figure 5C) revealed varying degrees of developmental inhibition in larvae fed transgenic leaves compared with the WT controls. Lines n19a and n19b exhibited similar inhibitory trends, maintaining stable molting indices initially before rising rapidly by day 12. DB16 and pb8 showed milder effects, with molting indices remaining below WT until pupation; the average difference did not exceed 0.82 ± 0.24. All transgenic lines delayed pupation: n19a, n19b, DB16, and pb8 extended the time to pupation by 8, 8, 4, and 2 days respective to WT (14 days), while pb9 and DB7 again failed to pupate. Regarding molting index inhibition against 3rd-instar larvae, pb vector lines performed best, followed by n5 vector lines, whereas n19 vector lines again exhibited relatively weaker effects.

#### 2.4.2. Analysis of Resistance Against *P. versicolora* in Transgenic Lines

**Figure 6:**
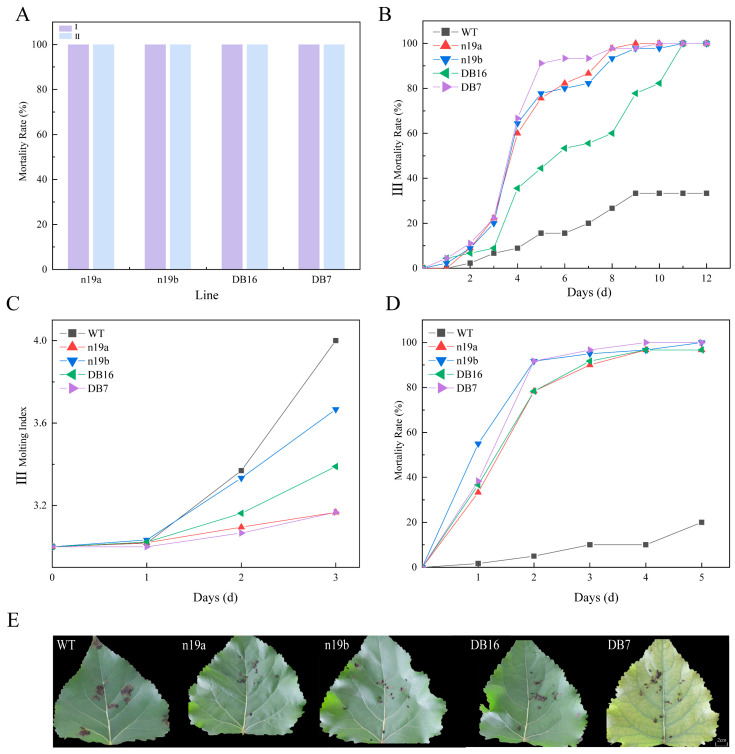
Insect resistance of transgenic lines against *P. versicolora*. (**A**) Corrected mortality of 1st- and 2nd-instar larvae across different lines. Roman numerals I–II denote the larval instar stage. (**B**) Mortality dynamics of 3rd-instar larvae. (**C**) Molting index dynamics of 3rd-instar larvae (pupal stage counted as 4th instar, adult as 5th instar). (**D**) Mortality dynamics of adult. (**E**) Leaf consumption by 3rd-instar larvae after 6 h at 4 days. The scale bar is approximate; the figure is presented as a schematic representation.

The Cry1Ac protein is specifically toxic to Lepidoptera insects, whereas Cry3A targets Coleopterans [31]. Therefore, this study selected four lines carrying the *Cry3A* gene for resistance analysis against *P. versicolora*. All transgenic lines demonstrated strong lethal effects on larvae at different instar stages (Figure 6A): 100% mortality was achieved for both 1st- and 2nd-instar larvae within one day of feeding.

Lethal effects on 3rd-instar larvae (Figure 6B) showed over 78% mortality within the first two days, indicating rapid toxicity. After day 2, mortality progression gradually slowed. By day 5, lines n19b and DB7 again reached 100% mortality, while n19a and DB16 both reached 96.67%. Leaf consumption assessed after 6 h of feeding on day 4 (Figure 6E) showed that WT leaves sustained the most severe damage, whereas leaves from the transgenic lines remained relatively intact.

Analysis of molting index changes in 3rd-instar larvae fed transgenic lines (Figure 6C; pupal stage counted as 4th instar, adult as 5th instar) revealed developmental inhibition across all lines, with differential effects emerging from day 2. Specifically, n19a and DB7 lines showed stronger developmental inhibition than n19b and DB16 lines.

Adults *P. versicolora* mortality also varied among the transgenic lines (Figure 6D). After two days of feeding, mortality ranged from 78.33% and 91.67%, with all lines showing similar lethal trends characterized by gradual increases after day 2. By day 5, lines n19a and DB7 reached 100% mortality, whereas n19b and DB16 achieved 96.67%.

#### 2.4.3. Analysis of Resistance Against *A. glabripennis* in Transgenic Lines

**Figure 7:**
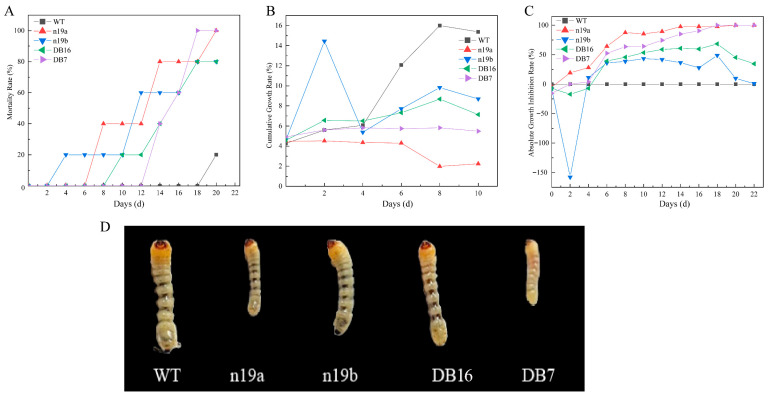
Resistance of transgenic lines against *A. glabripennis* larvae. (**A**) Larval mortality over time across treatment groups. (**B**) Inhibition of cumulative growth rate by different lines. (**C**) Absolute growth inhibition rate of larvae across treatment groups. (**D**) Larval development after 10 days of treatment in different lines.

Significant differences in lethal efficacy against *A. glabripennis* larvae were observed among transgenic lines (Figure 7A). Specifically, lines DB7 and n19a achieved 100% final mortality, whereas n19b and DB16 reached 80%. The mortality trends showed that n19b exhibited an early onset but plateaued later, while DB7, despite a later start, showed rapid progression toward the end of the assay.

In contrast, the WT group showed continuously increasing growth rates with marked acceleration in later stages; all transgenic lines exhibited varying degrees of growth inhibition (Figure 7B). Lines n19a and DB7 showed strong inhibition, whereas n19b and DB16 displayed dynamic fluctuations during specific phases.

In contrast, the WT group maintained a consistently low inhibition rate near zero; all transgenic lines displayed varying degrees of growth inhibition and differed in their dynamic patterns and persistence (Figure 7C,D). Lines n19a and DB7 showed sustained and stable growth inhibition with the strongest effects. Although n19b showed some inhibition, its efficacy fluctuated over time, while the inhibitory effect of DB16 gradually weakened as development progressed. Against *A. glabripennis*, the n19 vector lines again performed the best, whereas the n5 vector lines were relatively less effective.

### 2.5. Transcription Response of P. versicolora Larvae to Bt Toxin

After quality control, which included the removal of sequencing adapters, contaminants, and low-quality reads, a total of 60.07 Gb of clean data were obtained from the larval transcriptomes. The sequencing error rate was 0.01%, while the GC content ranged from 44.24% to 45.59%, and Q30 values were between 92.89% and 93.81% (Supplementary Table S5), confirming that the larval transcriptome sequencing data were suitable for subsequent analysis.

#### 2.5.1. Differential Gene Identification and Hierarchical Cluster Analysis in Larvae

**Figure 8:**
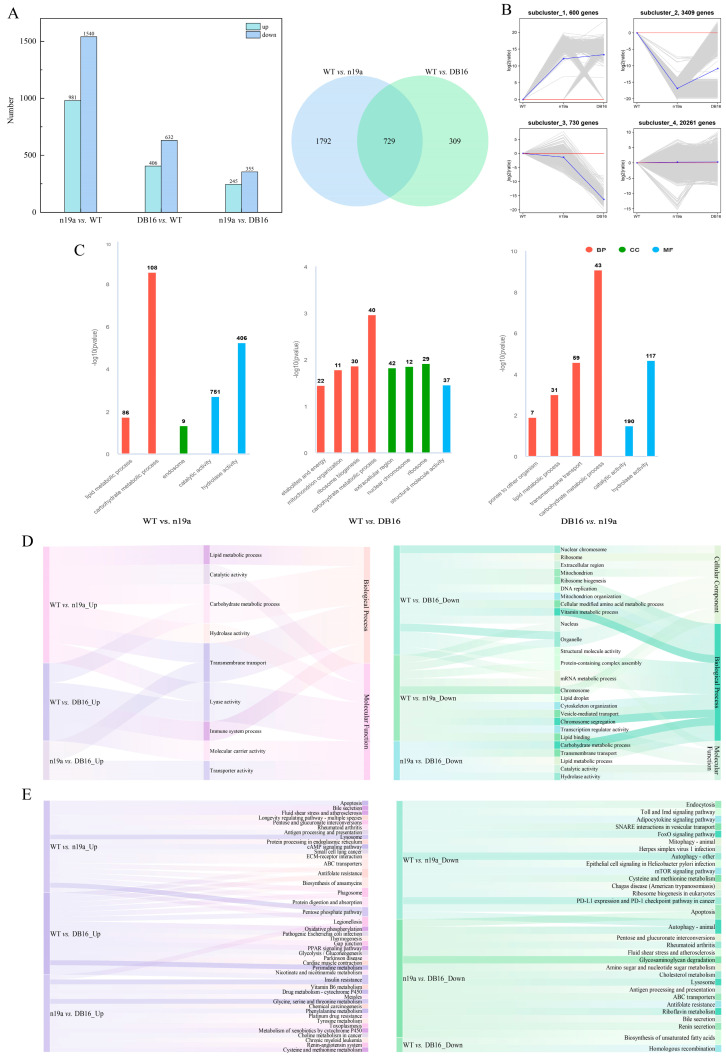
Identification, clustering, and functional enrichment analysis of differentially expressed genes. (**A**) Number of differentially expressed genes. (**B**) Hierarchical clustering of DEGs. (**C**) GO term enrichment statistics. (**D**) GO functional enrichment map. (**E**) Significantly enriched KEGG pathways.

To investigate how Bt toxin inhibits larval development in *P. versicolora*, differentially expressed genes (DEGs) in the larvae were identified using the thresholds |log_2_(FoldChange)| ≥ 1 and padj ≤ 0.05, followed by quantitative analysis (Figure 8A). Comparing the n19a treatment group with the WT control (n19a vs. WT) identified 2521 DEGs, including 1540 down-regulated and 981 up-regulated genes. The WT vs. DB16 comparison yielded 1038 DEGs, with 632 down-regulated and 406 up-regulated. Between the n19a and DB16 groups (n19a vs. DB16), 600 DEGs were detected, including 355 down-regulated and 245 up-regulated genes. Moreover, 729 DEGs were shared between the n19a vs. WT and WT vs. DB16 comparisons.

All DEGs from the larval transcriptomes of the two comparison groups were analyzed by K-means hierarchical cluster analysis (Figure 8B), yielding four distinct clusters. Subcluster_1 contained 600 genes, Subcluster_2 3409, Subcluster_3 730, and Subcluster_4 20,261. In Subcluster_1, gene expression was higher in both n19a and DB16 than in WT, with the DB16 group showing a continued upward trend. Subcluster_2 showed lower expression in larval n19a relative to WT, with partial recovery in DB16—though still below WT levels. Subcluster_3 displayed significantly reduced expression in DB16 compared with WT, while n19a remained relatively stable. Subcluster_4 maintained relatively stable expression across WT, n19a, and DB16, with only minor fluctuations around baseline, representing a stable gene set in larval.

#### 2.5.2. GO Functional Enrichment and KEGG Pathway Annotation of Larval Genes

Based on the GO database, larval DEGs were functionally annotated and then classified (Figure 8C). DEGs from the n19a vs. WT comparison were associated with five subcategories across biological process (BP), cellular component (CC), and molecular function (MF) (over-represented *p*-value < 0.05), showing significant enrichment of hydrolase activity, carbohydrate metabolism, endosomal membrane transport, catalytic activity, and lipid metabolic processes. DEGs from the WT vs. DB16 comparison spanned eight subcategories within BP, CC, and MF, primarily enriched in carbohydrate metabolic processes, ribosomes, ribosome biogenesis, nuclear chromosomes, extracellular regions, mitochondrial organization, structural molecule activity, and generation of precursor metabolites and energy. DEGs from the n19a vs. DB16 comparison were distributed across six subcategories in the BP and CC domains, with significant enrichment in hydrolase activity, transmembrane transport, carbohydrate metabolic processes, lipid metabolic processes, defense response to other organisms, and catalytic activity. The n19a and DB16 treatment groups exhibited distinct patterns in the GO terms enriched by their respective larval DEGs (Figure 8D and Figure S5).

Functional annotation and pathway analysis of larval DEGs using the KEGG database (Figure 8E) revealed substantial differences in metabolic pathway annotation and enrichment across comparison groups. In the WT vs. n19a comparison, the n19a treatment group exhibited significant up-regulation of pathways, including lysosome (28 DEGs), cAMP signaling pathway (27 DEGs), ABC transporters (26 DEGs), and antifolate resistance (20 DEGs) pathways. This was accompanied by activation of immune-related pathways such as lysosomal function and antigen presentation.

In contrast, the WT vs. DB16 comparison showed only mild up-regulation in the larvae of ABC transporters (6 DEGs) and antifolate resistance (4 DEGs), but significant activation of energy metabolism pathways, including oxidative phosphorylation (9 DEGs) and thermogenesis (9 DEGs). Down-regulated pathways in the larvae were primarily enriched in cellular senescence (7 DEGs) and ribosome (7 DEGs), indicating a shift toward perturbed energy metabolism and cellular senescence. This suggests that larvae in this treatment group adapt to toxin-induced stress by reprogramming energy metabolic pathways.

In the n19a vs. DB16 comparison, down-regulated pathways in larvae were mainly associated with lysosome (20 DEGs) and pentose-glucuronate interconversions (13 DEGs), while up-regulated pathways were dominated by tyrosine metabolism (5 DEGs). This reflects core differences in cellular degradation, metabolic conversion, and infection response between the two lines. Furthermore, pathways such as ABC transporters and apoptosis were consistently enriched across multiple comparisons in larval transcriptomes, suggesting their roles as conserved regulatory nodes under different treatments.

An in-depth analysis of these key pathways reveals their biological significance: the up-regulation of lysosomal pathways indicates an enhanced cellular capacity for the degrading and processing of foreign toxins; the activation of ABC transporters reflects increased cellular efflux of xenobiotics; and the reprogramming of energy–metabolism pathways shows how insects redistribute energy resources to cope with toxin stress. These coordinated changes across multiple pathways collectively constitute the molecular defense network of insects against Bt toxins.

#### 2.5.3. Transcription Factor Analysis in Larvae

**Figure 9:**
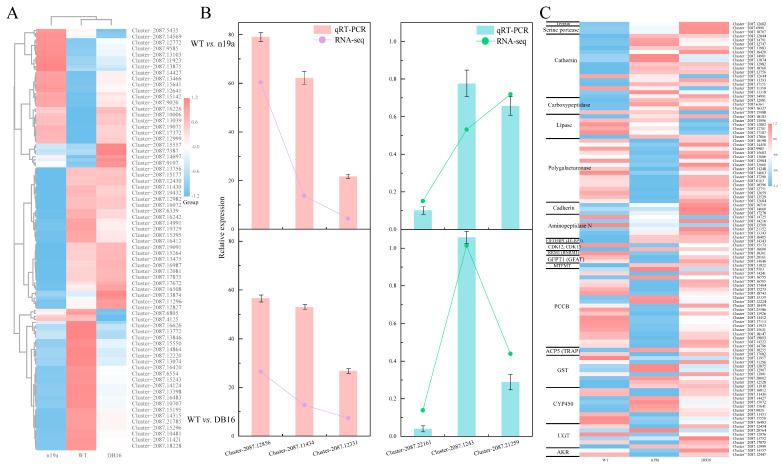
Analysis of transcription factors, qRT-PCR validation and differential gene expression. (**A**) Heatmap of transcription factor expression. Gene expression levels are shown in fragments per kilobase of transcript per million mapped reads (FPKM). (**B**) qRT-PCR validation. (**C**) Heatmap of differentially expressed genes related to development and Bt response.

Changes in transcription factor expression patterns can influence levels of downstream target genes (Figure 9A and Supplementary Table S6). In the WT vs. n19a comparison, 107 transcription factors were differentially expressed in larvae (30 up-regulated, 77 down-regulated), belonging to 23 families—primarily *zf-C2H2* (43), *THAP* (12), *ZBTB* (8), *Homeobox* (6), *MYB* (5), *HTH* (4), *HMG* (4), and *bHLH* (4). The WT vs. DB16 comparison yielded 25 differentially expressed transcription factors (10 up-regulated, 15 down-regulated) from 12 families, mainly *zf-C2H2* (7), *MYB* (4), and *THAP* (3). The n19a vs. DB16 comparison identified six larval transcription factors (four up-regulated, two down-regulated) from five families, predominantly *zf-C2H2* (2). Across all comparisons, down-regulated transcription factors outnumbered up-regulated ones. These expression patterns suggest that *P. versicolora* larvae modulate gene expression through differential transcription factor activity as part of their immune responses to Bt toxin.

#### 2.5.4. qRT-PCR Validation of Larval Gene Expression

Six larval genes were randomly selected for qRT-PCR to validate RNA-seq results. Comparison between qRT-PCR data and larval transcriptome sequencing data (Figure 9B) showed consistent expression patterns for all six genes across both analytical groups. The strong agreement between the two methods confirms the reliability of the larval transcriptomic data generated in this study.

#### 2.5.5. Screening of Larval Development and Bt-Related Genes in *P. versicolora*

Using differential gene IDs and NCBI annotations, we identified larval DEGs associated with Bt toxin mechanisms of action, detoxification enzymes, and larval development in *P. versicolora* (Table 2 and Table S7). Based on FPKM values, an expression heatmap (Figure 9C) was generated for the 108 selected larval DEGs, showing expression differences in Bt-response-related genes after larvae consumed the two transgenic lines compared with the control.

##### Proteases Involved in Bt protoxin Activation and Digestion in Larvae

Trypsin, a key digestive enzyme, plays an essential role in Bt protoxin in the larval midgut. In this study, after *P. versicolora* larvae consumed poplar leaves containing Cry3A toxin, the expression of protoxin activation-associated enzyme genes was altered. Among the DEGs in WT vs. n19a, one trypsin-related gene was up-regulated. In the WT vs. DB16 comparison, one trypsin-related gene was down-regulated.

After insects ingest food, multiple digestive hydrolases participate in the digestion and absorption within the larval midgut. Beyond the trypsin-like proteases already mentioned, various other enzymes contribute to these processes. In the WT vs. n19a DEG set, we identified 2 serine protease-related genes (both up-regulated), 16 cathepsin-related genes (14 up-regulated, 2 down-regulated), 4 carboxypeptidase-related genes (3 up-regulated, 1 down-regulated), 6 lipase-related genes (2 up-regulated, 4 down-regulated), and 15 polygalacturonase-related genes (all up-regulated). In the WT vs. DB16 comparison, we detected 1 serine protease-related gene (up-regulated), 1 lipase-related gene (down-regulated), and 3 polygalacturonase-related genes (all up-regulated). No cathepsin- or carboxypeptidase-related genes were found in this comparison. After consuming Bt toxin-containing poplar leaves, *P. versicolora* larvae showed altered expression of multiple digestive enzymes in their midgut, suggesting their potential involvement in immune responses to the toxin.

##### Differential Genes Associated with Potential Bt toxin Receptor Proteins in Larvae

The toxic peptides released by Bt toxins must bind to specific receptors on larval midgut cells to exert their effects. After *P. versicolora* larvae consumed poplar leaves containing Cry3A toxin, significant changes occurred in genes encoding potential toxin-binding receptors. In the WT vs. n19a DEGs, we identified three toxin-binding receptor-related genes (all up-regulated) and six aminopeptidase N-related genes (four up-regulated, two down-regulated). In the WT vs. DB16 DEGs, we found one cadherin gene (up-regulated) and one aminopeptidase N gene (up-regulated). These changes in larval receptor genes may modulate Bt toxin action pathways.

##### Genes Associated with Detoxification Enzyme Systems in Larvae

Detoxification enzyme systems, such as cytochrome P450 monooxygenases (CYPs) and glutathione S-transferases (GSTs), play crucial roles in insect metabolic resistance to xenobiotics. UDP-glucuronosyltransferases (UGTs) are key metabolic and detoxification enzymes that transform xenobiotics and help maintain internal homeostasis, while aldo-keto reductases (AKRs) catalyze aldehydes reduction and are essential for detoxifying aldehyde compounds and supporting normal physiological functions. After *P. versicolora* larvae consumed transgenic poplar diets, there was a significant occurrence of the expression of detoxification enzyme-related genes. From the WT vs. n19a DEGs, we identified eight glutathione S-transferase genes (three up-regulated, five down-regulated), nine cytochrome P450 genes (five up-regulated, four down-regulated), five UDP-glucuronosyltransferase genes (three up-regulated, two down-regulated), and two aldo-keto reductase genes (both up-regulated). From the WT vs. DB16 DEGs, we detected three glutathione S-transferase genes (one up-regulated, two down-regulated), one cytochrome P450 gene (up-regulated), and two UDP-glucuronosyltransferase genes (one up-regulated, one down-regulated). No aldo-keto reductase genes were identified in this comparison.

##### Differentially Expressed Genes Associated with Larval Growth and Development

After consuming transgenic poplar leaves, *P. versicolora* larvae showed significant changes in the expression of genes related to growth and development. In the WT vs. n19a DEGs, we identified 1 eukaryotic translation initiation factor 4E-binding protein gene (down-regulated), 2 cyclin-dependent kinase 12/13 genes (1 up-regulated, 1 down-regulated), 1 ribonucleoside-diphosphate reductase M1 gene (up-regulated), 2 glutamine-fructose-6-phosphate transaminase genes (both up-regulated), 2 methionyl-tRNA formyltransferase genes (both down-regulated), 20 propionyl-CoA carboxylase beta chain genes (16 up-regulated, 4 down-regulated), and 2 lysosomal acid phosphatase genes (1 up-regulated, 1 down-regulated). In the WT vs. DB16 DEGs, we detected one eukaryotic translation initiation factor 4E-binding protein gene (down-regulated), one ribonucleoside-diphosphate reductase M1 gene (up-regulated), and two propionyl-CoA carboxylase beta chain genes (both up-regulated). No genes related to cyclin-dependent kinases 12/13, glutamine-fructose-6-phosphate transaminase, methionyl-tRNA formyltransferase, or lysosomal acid phosphatase were found in this comparison.

## 3. Discussion

Since its emergence, transgenic technology has been widely adopted in forest tree breeding. An increasing number of tree species have acquired traits such as insect resistance, cold tolerance, drought tolerance, and salt-alkali tolerance through molecular breeding [32]. The *Bt* gene, derived from *Bacillus thuringiensis*, encodes insecticidal crystal proteins. Numerous studies have reported successful expression of *Bt* insecticidal proteins in plants, with excellent pest control efficacy. For example, Dimase et al. [33] conducted selective insect feeding experiments with corn under greenhouse conditions and found no live fall armyworm (*Spodoptera frugiperda*) larvae or plant damage on *Bt*-transgenic corn, confirming strong insect resistance. Similarly, research on *Bt*-transgenic Nanlin 895 poplar showed that the introduction of *Bt* genes effectively suppressed the growth of *H. cunea* and exhibited notable insecticidal efficacy [34]. However, different crystal proteins have distinct insecticidal specificities. Because many target traits are regulated by multiple genes acting together, single-gene transformation often fails to achieve the desired breeding outcomes. As a result, many researchers have shifted their focus to multigene expression vectors, which can carry several target genes simultaneously, enabling recipient plants to acquire broader insect resistance spectra [35,36]. This study employed six transgenic poplar 107 lines derived from three distinct vector constructs: the pb construct (lines pb8, pb9; single *Cry1Ac*), the n19 construct (lines n19a, n19b; multigene, reverse-oriented), and the n5 construct (lines DB7, DB16; multigene, forward-oriented). Comparative analyses focused on the differences in insect resistance and gene expression patterns among these three Bt gene configurations.

### 3.1. Variations in Exogenous Gene Expression and Insect Resistance Among Transgenic Lines

Transgenic *Bt* poplar 107 lines (pb8/pb9, n19a/n19b, DB7/DB16) showed distinct patterns in *Bt* gene expression levels. PCR confirmed successful integration of the target genes in all six transgenic lines. qRT-PCR and ELISA results indicated that both transcript abundance and protein accumulation of Cry3A were significantly higher than those of Cry1Ac. This trend is consistent with findings by Ren et al. [30], which also indicate stronger expression dominance of *Cry3A* in dual-gene vector systems. Despite higher *Cry3A* expression levels, the single-gene *Cry1Ac*-transformed line pb9 exhibited greater lethality against the lepidopteran insect *H. cunea* than most dual-gene lines. The n19-derived lines (n19a/n19b) characterized here exhibited strong broad-spectrum resistance to *H. cunea* and *P. versicolora*, and the pb-derived lines (pb8/pb9) showed targeted activity against *H. cunea.* This result agrees with the observation of Cao et al. [37], indicating that insecticidal efficiency depends not only on expression levels but also on specific toxic mechanisms targeting particular pests.

In dual-*Bt* gene lines (n19a, n19b, DB16), Cry1Ac toxin protein levels were generally lower than in the single-gene line pb9, possibly due to transcriptional competitive inhibition from multigene co-expression [38]. Potential mechanisms may include transcriptional conflicts caused by RNA polymerase stalling at promoter [39], reduced mRNA stability in 3′UTRs (e.g., AU-rich elements, AREs) [40], and impaired translation initiation due to secondary structures in Kozak sequences [41]. Furthermore, multigene expression might activate endogenous protein degradation pathways such as ubiquitination or phosphorylation, thereby reducing toxin protein accumulation and activity [42]. However, the precise molecular basis requires further validation. Future studies should focus on whether position effects influence promoter activity, investigate potential RNA-level interference, and compare mRNA stability between single and dual-gene contexts. Such work would help clarify the suppression mechanism and guide the design of optimal multi-gene vectors.

### 3.2. Transgenic Lines Exhibited Differential Insecticidal Efficacy Against the Two Coleopteran Species

To clarify the specificity of the Cry3A protein against different target insects, this section compares the effects of six transgenic poplar 107 lines (representing three vector types) on two coleopteran species: *P. versicolora* and *A. glabripennis*. The results show that the same transgenic lines caused 100% mortality in both larvae and adults of *P. versicolora* within the experimental period, whereas for *A. glabripennis* maximum mortality reached only 80%, with a longer time required to achieve high lethality. These findings indicate that *P. versicolora* is more susceptible to the *Cry3A* than *A. glabripennis*.

Regarding developmental impacts, all six transgenic lines exhibited distinct inhibition patterns between the two insect species. Larvae of *P. versicolora* fed transgenic leaves showed an 8.33~20.83% reduction in molting index compared with the wild-type (WT) group, indicating significantly delayed development. In contrast, growth inhibition rates for *A. glabripennis* larvae varied widely across six lines, ranging from 4.3% to 141.22%, reflecting substantial variability in the growth-suppressive effects of different transgenic lines. Because these conclusions are based on a limited number of lines, future studies involving more transformation events are needed to confirm the observed patterns.

The observed differences in toxicity may stem from three factors: First, insect body size—*P. versicolora* is of a smaller size, leading to higher toxin intake per unit body weight and thus more pronounced toxic effects. Second, feeding behavior—*P. versicolora* feeds directly on leaves, allowing more direct and efficient toxin ingestion than *A. glabripennis* larvae, which consume wood debris [43]. Third, physiological differences between the two insect species may directly influence Cry3A binding efficiency and toxicity [44].

In summary, by comparing the responses of two coleopteran species to the Cry3A protein, this study reveals clear differences in the toxin’s effects between target insects. These findings provide a theoretical basis for understanding the mode of action of Bt proteins and for the precision breeding of transgenic insect-resistant trees.

### 3.3. Comparative Analysis of Insect Resistance Conferred by Different Vector Constructs

The comparative analysis of insect resistance performance across the three vector types (single-gene *pb* vector vs. multigene *n19* and *n5* vectors) revealed that vector architecture is a key determinant shaping resistance trait.

Regarding lepidopteran pests, the pb vector accumulated lower Cry1Ac protein levels than some multigene lines, it caused faster mortality in both 1st and 3rd instar *H. cunea* larvae, demonstrating specialized efficacy against this lepidopteran pest [45]. This outcome indicates that single-gene vectors may achieve efficient toxin utilization through more specialized expression regulation mechanisms, underscoring the complexity that “high expression does not necessarily equal high activity.”

In contrast, for coleopteran pests, the multigene vectors n19 (reverse-oriented) and n5 (forward-oriented), represented by four transgenic lines, showed no significant differences in lethal efficacy against *P. versicolora* and *A. glabripennis*, indicating that gene arrangement and orientation have limited influence on resistance to these two coleopteran pests. However, both multigene vectors displayed dose–response relationships between toxin protein content and mortality [46], along with broader-spectrum insect resistance characteristics. A notable and complex pattern emerged concerning pest developmental stages. The insect resistance efficacy of different vector types varied across pest developmental stages. All vectors showed LT_50_ values of 24–48 h against 3rd-instar *P. versicolora* larvae, but this extended to 72–144 h for adults. The n19 vector performed best against early instars, while the n5 vector showed relatively stronger inhibition of adults. This vector-specific, stage-dependent resistance pattern—”greater efficacy against early instars than adults”—aligns with the findings of Zhang Qi et al. [47] and may support precision control strategies tailored to specific insect stages. From a mechanistic perspective, the stronger resistance in later instars may be associated with several factors: more developed peritrophic membranes in the midgut, elevated activity of detoxification enzymes (CYP450, glutathione), and enhanced antioxidant and excretion functions [48,49,50]. These physiological differences likely alter the efficiency with which toxin proteins expressed by different vector types act within the insect body, ultimately shaping the observed resistance outcomes.

In summary, this comparative analysis indicates that vector type is a critical factor shaping the insect control efficacy of transgenic plants. Single-gene vectors exhibit specialized, high efficacy against specific target pests, whereas multigene vectors provide broader protection across insect orders. It should be noted that the conclusions herein are primarily inferred from the assessment of insect resistance in the six transgenic lines examined. Future vector design should select appropriate types based on the desired resistance spectrum and target pest characteristics to achieve optimal insect control outcomes. To improve the reliability of these findings, subsequent studies should expand the scale of transgenic line screening to account for potential variations due to factors such as transgene insertion sites and copy numbers.

It should be noted that the conclusions of this study, derived from six transgenic lines, have inherent limitations. Variations in insect resistance phenotypes among lines due to factors such as insertion sites and copy numbers may influence the accurate assessment of differences between vector types. Subsequent studies should therefore expand the scale of line screening to improve the reliability of findings.

### 3.4. Transcriptomic Response of P. versicolora Larvae to Bt Toxin

**Figure 10:**
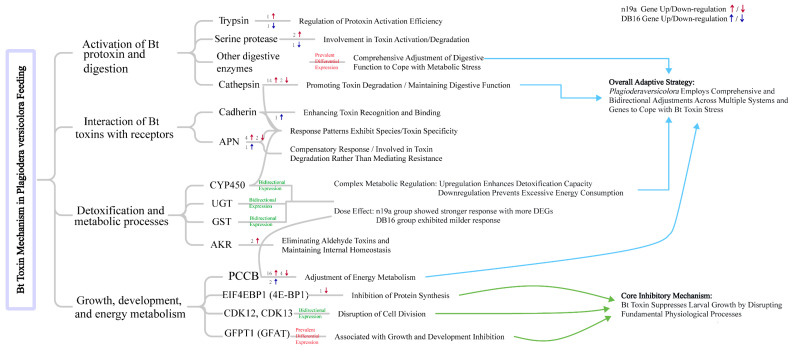
Transcriptional response mechanisms in *P. versicolora* after ingestion of Bt toxins. Blue arrows: Connect the response logic across different systems, leading to the summary of the overall adaptive strategy; Green arrows: Consolidate the inhibitory effects related to growth and development, pointing to the summary of the core inhibitory mechanism.

Transcriptome analysis of *P. versicolora* larvae fed on high-resistance n19a versus low-resistance DB16 lines revealed a complex molecular regulatory network underlying larval responses to Bt toxin stress (Figure 10). Regarding toxin activation and metabolism, trypsin genes showed distinct expression patterns between treatment groups—significantly up-regulated in n19a but down-regulated in DB16. These differences may correspond to variations in toxin expression and potency among the six transgenic lines [30,51]. Serine protease genes were up-regulated in both groups, suggesting their potential role in toxin activation or degradation [52]. Notably, most cathepsin genes were up-regulated in the n19a group, consistent with response reported in western corn rootworm [53]. This indicates that coleopteran insects may increase the activity of such proteolytic enzymes to promote toxin degradation or maintain digestive function. Cross-species differences could arise from variations in feeding habits, midgut environments, and toxin types [54,55].

At the toxin recognition and binding level, cadherin genes were up-regulated in the DB16 group, which may enhance toxin binding efficiency to midgut epithelial cells and reflect active insect recognition of toxins [56,57]. Aminopeptidase N (APN)-related genes were predominantly up-regulated in both groups, possibly representing an initial compensatory response to toxin-induced damage or functional differentiation among family members involved in toxin degradation [58,59].

Regarding detoxification metabolism, key enzymes such as glutathione S-transferase (GST) and cytochrome P450 (CYP450) showed bidirectional expression patterns. In the n19a group, reduced expression of some detoxification enzymes may reflect system overload under high toxin concentrations, while relatively stable expression in the DB16 group suggests that detox mechanisms operate effectively under lower toxin pressure [60,61]. Notably, aldo-keto reductase (AKR) genes were exclusively up-regulated in the n19a group, indicating their potential role in clearing toxic aldehydes under high toxin stress to maintain cellular homeostasis [62].

In growth and development regulatory pathways, genes showed especially pronounced changes in the n19a group. Down-regulated eukaryotic translation initiation factors suppressed protein synthesis, disrupted cyclin-dependent kinase expression interfered with normal cell division cycles, and altered expression of the PCCB gene indicated metabolic reprogramming via adjustments in energy metabolism to cope with toxin stress [63,64,65]. Together, disruption of these key pathways impaired larval development, providing a molecular explanation for the experimentally observed 8.33~20.83% reduction in molting index and the associated growth inhibition.

In summary, *P. versicolora* employs multi-level transcriptional reprogramming that integrates toxin metabolism, cellular recognition, detoxification responses, and developmental regulation to cope with toxin stress. These findings offer molecular insights into the differential responses to Bt toxin between the two lines, and provide an intrinsic explanation for its higher susceptibility compared with *A. glabripennis*. Further studies involving additional transgenic lines will help determine whether these transcriptomic patterns are consistently linked to specific vector architectures.

### 3.5. Research Summary and Future Directions for Application

This study systematically compares the insect resistance efficacy of transgenic poplar lines carrying three distinct vector constructs. The key finding is that vector architecture significantly shapes both the level and the spectrum of insect resistance: single-gene vectors provide high efficiency against specific pests, whereas multigene vectors deliver broader protection. These results offer a practical framework for future precision breeding, enabling the selection of optimal vector systems according to the target pest spectrum in specific regions. To further advance the mechanistic understanding of insect–Bt interactions, future research should include functional validation of key candidate genes identified through transcriptomic analysis. Applying gene-knockout techniques in target insect species would help establish causal links between specific gene expression changes and the observed insect resistance phenotypes. This study is limited to six transgenic lines. Unquantified variation in transgene copy number may partly explain the observed differences in *Bt* gene expression and insect resistance among these lines. Therefore, the findings are specific to the characterized lines. Future work should include copy number verification to further clarify the underlying expression-regulation mechanisms.

Before any field deployment, cultivation of such transgenic trees would be subject to rigorous biosafety regulations and risk assessments, in line with standards for genetically modified organisms (GMOs). A key risk assessment focus is transgene flow to wild relatives. To mitigate this, containment measures—such as using sterile cultivars or maintaining spatial isolation from compatible wild poplar populations—would be essential prior to environmental release. Thus, while this research demonstrates considerable potential for targeted forest pest management, its practical application must be guided by a responsible, science-based regulatory framework to ensure ecological safety.

## 4. Materials and Methods

### 4.1. Plant Material

Based on previous laboratory results, six transgenic 107 poplars and non-transgenic wild-type 107 poplar (labeled as WT) controls were obtained from the specialized nursery of Hebei Agricultural University. These lines were selected for a first-time parallel comparative analysis of insect resistance according to their vector architectures. Using the mortality of 1st-instar *H. cunea* larvae after 10 days of feeding on transgenic leaves, lines with less than 60% mortality were classified as low-resistant and those with over 80% mortality as high-resistant. The six transgenic lines comprised the following: pb9 (high-resistance) and pb8 (low-resistance) [28], generated with the pb vector carrying *Cry1Ac*; n19a (high-resistance) and n19b (low-resistance) [29], generated with the n19 vector carrying *Cry1Ac* and *Cry3A* in reverse orientation; DB7 (high-resistance) and DB16 (low-resistance) [30], generated with the n5 vector carrying *Cry3A* and *Cry1Ac* in tandem. Although n19a and n19b were initially produced by other laboratory members, this study presents their first systematic insect resistance evaluation. The arrangement of genes on each vector is shown in Figure 1.

Three forest pests were used in this study: *H. cunea*, obtained from the state-owned forest farm in Luannan County, Hebei Province; *P. versicolora*, collected from the Experimental Farm of Hebei Agricultural University; and *A. glabripennis*, provided by the Forest Protection Laboratory of the Hebei Agricultural University.

### 4.2. PCR and qRT-PCR Detection of Exogenous Genes in Transgenic Lines

Genomic DNA was extracted from leaves of transgenic lines and wild-type control using the CTAB method. PCR amplification of exogenous *Bt* genes was performed with the Cowin Biotech 2× Es Taq MasterMix (Dye) PCR kit (Cowin Biotech Co., Ltd., Taizhou, Jiangsu, China). Plasmid DNA containing *Cry1Ac* and *Cry3A* served as the positive control, and DNA from non-transgenic plants was used as the negative control.

During the 2024 growing season, mature leaves from all transgenic lines and wild-type controls were collected, flash-frozen in liquid nitrogen, and stored at −80 °C until use. Total RNA was extracted from the leaves using the Plant Polysaccharide & Polyphenol Total RNA Extraction Kit (Zhangjiakou Sinuo Biotechnology, Zhangjiakou, China). Genomic DNA removal and first-strand cDNA synthesis was carried out using the One-Step gDNA Removal and cDNA Synthesis SuperMix (Beijing TransGen Biotech, Beijing, China). qPCR primers specific to *Cry1Ac* and *Cry3A* (designed in our previous work) were used for amplification (Supplementary Table S8) [30]. The PCR products were purified, quantified, and serially diluted in 10-fold steps to generate standard curves. Absolute quantitative PCR was performed on standard samples and cDNA was performed with PerfectStart Green qPCR SuperMix (Beijing TransGen Biotech) on the Agilent Technologies Stratagene Mx3005P Real-Time PCR (Agilent, Santa Clara, CA, USA) (20 μL reaction volume, three biological replicates per sample). Transcript abundance was analyzed by the 2^−ΔΔCt^ method [66] to calculate relative expression, and all expression values were log_10_-transformed.

### 4.3. Analysis of T-DNA Insertion Sites and Bt Toxin Detection in Transgenic Lines

Fresh leaves from four transgenic lines (n19a, n19b, pb8, and pb9) were collected, and genomic DNA was extracted using the CTAB method. Whole-genome resequencing was conducted on the Illumina platform by Guangzhou Gidio Biotechnology Co., Ltd. (Guangzhou, China), following their standard procedures. The DB7 and DB16 lines had been sequenced previously in the laboratory [30]. Vector-specific primers (Supplementary Table S9) were designed based on the vector sequence. Depending on insertion orientation, these primers were paired with corresponding genome-specific primers to amplify genomic DNA from each line, thereby validating T-DNA insertion sites and orientations. Genomic regions spanning 20 kb upstream and downstream of each insertion site were extracted from the genome and annotated for gene function using the InterPro database.

Bt toxin proteins in each line were quantified with the Fankewei Plant Bt Toxin ELISA Kit according to manufacturer’s instructions. Standard curves were generated by plotting absorbance against known standard concentrations. The curves for Bt1 and Bt3 were y = 5.7442x + 0.1703 (R^2^ = 0.9996) and y = 20.972x − 2.5024 (R^2^ = 0.9977), respectively, where x represents absorbance and y represents concentration. Three biological replicates were performed; OD_450_ values were measured with a microplate reader, and toxin protein concentrations were calculated using the linear regression of the standard curves. Statistical analysis was conducted by one-way ANOVA using SAS 9.4.

### 4.4. Insect Resistance Assessment of Transgenic Lines—Insect Feeding Bioassay/Calculation Methods

(1) *H. cunea*

Freshly excised leaves from six transgenic lines containing the *Cry1Ac* (n19a, n19b, pb8, pb9, DB16, DB7) and wild-type (WT) controls were used. For each line and WT, leaves were collected from three independent, vegetatively propagated trees (three biological replicates), each serving as an experimental unit.

To standardize insect load, each replicate (a sealed rearing box containing one leaf) received 40 1st-instar and 10 3rd-instar larvae, maintained at room temperature. Leaves were replaced every 48 h; petioles were kept in 2 mL centrifuge tubes filled with water to maintain humidity. Larval mortality and the instar stage of surviving larvae were recorded every 48 h. The total eclosion rate was calculated after adult emergence.

(2) *P. versicolora*

Detached leaves from four transgenic lines containing the *Cry3A* (n19a, n19b, DB16, DB7) and WT controls were used. Tests were performed on four developmental stages: 1st-, 2nd-, and 3rd-instar larvae, and adults. For each treatment and stage, three biological replicates (leaves from three different trees) were established.

Each larval replicate contained 20 insects; each adult replicate contained 15 insects. Mortality, survival, and instar stages were recorded every 24 h. Leaves were rehydrated every 12 h and replaced every 48 h. Observations continued until all insects had either eclosed or died. Calculation followed the formulas above.

(3) *A. glabripennis*

The tested lines were identical to those described above. Stems and leaves from each transgenic line and WT (from three individual trees per line) were used. The diet consisted of one-year-old cutting stems and leaves, homogenized using a multifunctional grinder, portioned into 10 mL centrifuge tubes, and stored at −80 °C until use.

Five larvae were reared per line per biological replicate, with three replicates per line (totaling 15 larvae per line). Each larva was individually cultured in a 10 mL centrifuge tube to prevent cannibalism and control individual growth conditions. The diet was replaced every 48 h, and larval body weight was recorded at each diet change. After 20 days of feeding, the following parameters were calculated.

Calculation Methods:

Lethality—The following formulas were used to calculate the resistance indices:

Corrected mortality (%) = (Mortality in transgenic line − Control mortality)/(1 − Control mortality) × 100

Lethality rate (%) = (Number of dead larvae/Total number of larvae) × 100

Instar-specific lethality rate (%) = (Number of dead larvae at a specific instar/Total number of larvae) × 100

Pupation rate (%) = (Number of pupae/Initial number of insects) × 100

Total eclosion rate (%) = (Number of successfully emerged adults/Initial number of insects) × 100

Molting Index = Σ (Instar number × Number of larvae at that instar)/Total number of surviving larvae

Absolute growth inhibition rate (%) = (Weight gain of WT − Weight gain of Treatment group)/Weight gain of WT × 100

Cumulative growth rate (%) = (Final weight − Initial weight)/Initial weight × 100

### 4.5. Transcriptome Sequencing and qRT-PCR Validation of P. versicolora Larvae

Based on insect feeding assay results, 3rd-instar *P. versicolora* larvae showing significant mortality were selected for transcriptome analysis (Total RNA was extracted using the Tiangen DP424 kit, Tiangen Biotech Co., Ltd., Beijing, China). Fresh leaves from Cry3A-containing lines (n19a, DB16) and non-transgenic control (WT) lines were collected and used to feed third-instar larvae subjected to 12 h starvation pretreatment. Each experimental replicate included 30 insects, with three biological replicates per treatment. After 4 h of feeding on treated leaves, 8 larvae were randomly sampled from each replicate (24 larvae per treatment group) and pooled, resulting in nine sample groups for transcriptome sequencing analysis. After feeding, total RNA of the target larvae was extracted using the Tiangen DP424 kit following the manufacturer’s protocol. RNA quality was verified via NanoDrop 2000 (Thermo Fisher Scientific, Waltham, MA, USA) and Agilent 2100 Bioanalyzer (Agilent Technologies, Santa Clara, CA, USA), with an RNA integrity number (RIN) ≥ 7.0 required for library construction [67]. Strand-specific transcriptome library preparation was performed by Novogene (Beijing, China) following their standard protocol for insect samples [68,69], which included mRNA enrichment, fragmentation, cDNA synthesis, end repair, adapter ligation, size selection, and PCR amplification. The qualified library was sequenced on an Illumina NovaSeq 6000 platform to generate paired-end reads [69]. Library preparation and quality control were performed as follows: libraries were quantified using a Qubit 2.0 Fluorometer (Thermo Fisher Scientific, Waltham, MA, USA) and diluted to 1.5 ng/μL, with insert size verified by Agilent 2100 Bioanalyzer. Libraries with effective concentrations >1.5 nM were used for sequencing. Paired-end sequencing (150 bp) was performed on the Illumina platform. Raw sequencing data were quality-filtered using fastp with the following criteria: (1) removal of adapter-containing reads, (2) exclusion of reads with >10% unidentified nucleotides, and (3) elimination of reads where >50% of bases had Q-scores ≤ 20. Clean reads were aligned to the reference genome using DNAMAN V6. Following Trinity assembly, gene functions were annotated using seven major databases: Nr, Nt, Pfam, KOG, Swiss-Prot, KEGG, and GO. Gene expression levels were quantified in fragments per kilobase of transcript per million mapped reads (FPKM) and are presented in these units throughout the text and figures. Differentially expressed genes (DEGs) were identified with the thresholds of |log_2_FC| ≥ 1 and padj ≤ 0.05, yielding DEG sets for three comparison groups: n19a vs. WT, WT vs. DB16, and n19a vs. DB16. Enrichment analyses of GO functions and KEGG pathways were conducted using GOseq and KOBAS, respectively.

To validate the reliability of the transcriptome data, six differentially expressed genes (three up-regulated and three down-regulated) common to two comparison groups were randomly selected for qRT-PCR verification. The eukaryotic elongation factor EF1α gene was used as the reference gene (primer sequences for each gene are provided in Supplementary Table S10).

### 4.6. Statistical Analysis

Data processing and statistical analyses were performed using Microsoft Excel 2021 and SPSS 27.0.1. One-way analysis of variance (ANOVA) was applied to analyze the data for each treatment, followed by Duncan’s new multiple range test for significance testing (*p* < 0.05). Figures were generated using Adobe Illustrator 2022 and OriginPro 2021.

## 5. Conclusions

This study systematically evaluated insect resistance performance and mechanisms across six transgenic poplar lines. All lines successfully expressed Bt toxin proteins, with n5 vector lines showing multiple insertion sites while the others contained single-site insertion. Although the limited number of lines calls for cautious interpretation, consistent trends emerged: lines carrying single *Bt* genes exhibited particularly strong insecticidal activity against the lepidopteran pest *H. cunea*. Among the specific lines tested, multi-gene vector lines, though relatively less effective against *H. cunea*, significantly broadened the insect resistance spectrum, showing strong toxicity and developmental inhibition against both coleopteran insects—*P. versicolora* and *A. glabripennis.* Notably, the DB7 line displayed the broadest and strongest resistance against all three insect species.

Mechanism analysis revealed that the response of *P. versicolora* larvae to Bt toxin involves coordinated regulation of multiple genes, spanning key pathways such as protoxin activation, receptor binding, detoxification metabolism, and growth development. These findings not only clarify the complex physiological adaptation that insects employ against Bt toxins, but also provide an important theoretical foundation for the precision breeding of transgenic insect-resistant trees. Together, the results indicate that the rational selection of these characterized lines (multi-gene lines for broad resistance, single-gene lines for targeted control) can enable efficient insect-resistant breeding tailored to different pests, offering new technical pathways for integrated forest pest management.

## Figures and Tables

**Table 1:** T-DNA insertion positions in transgenic poplar lines.

Vector	Line	Chromosome	Insertion Site	Insertion Type	Orientation
n19	n19a	Chr2	4,814,040	Paired-end	Reverse
	n19b	Chr5	20,284,755	RB	Forward
pb	pb8	Chr10	2,683,280	LB	Forward
	pb9	Chr11	4,359,721	LB	Forward
n5	DB16	Chr1	30,504,409	Paired-end	Reverse
		Chr12	14,365,239	Paired-end	Tandem Repeat (Forward-Reverse)
		Chr17	13,926,822	Paired-end	Reverse
	DB7	Chr10	344,937	LB	Forward
		Chr10	21,796,805	RB	Reverse

**Table 2:** Genes related to growth, development, and Bt response in the transcriptome of *P. versicolora*.

Genes	WT vs. n19a			WT vs. DB16		
	Up	Down	Total	Up	Down	Total
Bt prototoxin activation and digestion-related protease						
Trypsin	1	0	1	1	0	1
Serine protease	2	0	2	1	0	1
Cathepsin	14	2	16	0	0	0
Carboxypeptidase	3	1	4	0	0	0
Lipase	2	4	6	0	1	1
Polygalacturonase	15	0	15	3	0	3
Potential Bt-binding receptors						
Cadherin	3	0	3	1	0	1
Aminopeptidase N	4	2	6	1	0	1
Detoxification enzymes						
GST	3	5	8	1	2	3
CYP450	5	4	9	1	0	1
UGT	3	2	5	1	1	2
AKR	2	0	2	0	0	0
Growth and development						
Eif4ebp1(4E-BP1)	0	1	1	0	1	1
CDK12,CDK13	1	1	2	0	0	0
RRM1(RNRM1)	1	0	1	1	0	1
GFPT1(GFAT)	2	0	2	0	0	0
MTEFMT	0	2	2	0	0	0
PCCB	16	4	20	2	0	2
ACP5(TRAP)	1	1	2	0	0	0

## Data Availability

The original contributions presented in this study are included in the article/Supplementary Materials. Further inquiries can be directed to the corresponding author(s).

## Supplementary Materials

The following supporting information can be downloaded at https://www.mdpi.com/article/10.3390/plants15010051/s1: Figure S1: PCR detection of exogenous genes in transgenic lines; Figure S2: RNA quality was assessed by 1.5% agarose gel electrophoresis; Figure S3: Analysis of the Cry1Ac gene by qRT-PCR; Figure S4: Analysis of the Cry3A gene by qRT-PCR; Figure S5: Significantly enriched KEGG pathways in each comparison group; Table S1: Transcription information; Table S2: Insertion site nearby gene function; Table S3: Genomic primers for insertion site validation; Table S4: Insertion site validation primer set; Table S5: Results of gene function annotation; Table S6 Transcription factor analysis; Table S7: DEGs identified in the transcriptome of *A. glabripennis* larvae in response to Bt toxin; Table S8: Gene-specific qPCR primer sequences; Table S9: Insertion site validation vector primer; Table S10: Transcriptome data qRT-PCR validation primers.
