# Supplementary material for: Comparative Analysis of Insect Resistance in Transgenic Populus × euramericana cv. Neva Expressing Dual Bt Genes from Different Sources
Source: Plants (Basel). 2025 Dec 23;15(1):51. doi: 10.3390/plants15010051 (PMC12787643; doi:10.3390/plants15010051)
Supplement: Supplementary file 1 [file plants-15-00051-s001.zip › plants-4008162-supplementary/Supplementart Appendix S1.pdf]

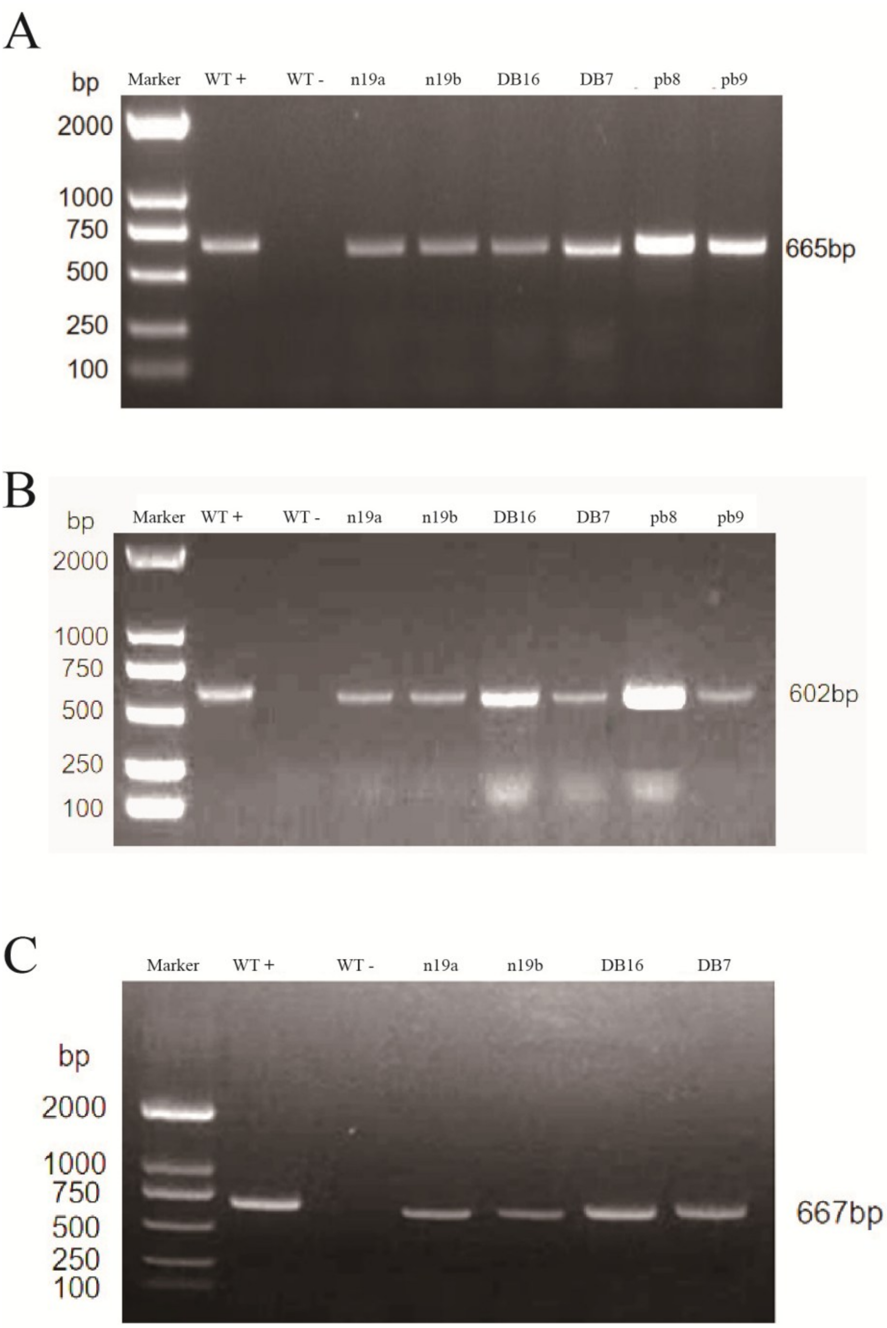

Figure S1. PCR detection of exogenous genes in transgenic lines. (A) Detection of the *nptII* gene in transgenic lines; (B) Detection of the *Cry1Ac* gene in transgenic lines; (C) Detection of the *Cry3A* gene in transgenic lines.

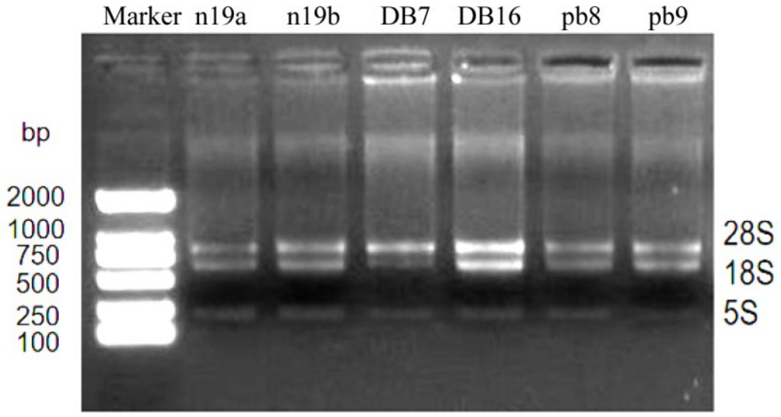

Figure S2. RNA quality was assessed by 1.5% agarose gel electrophoresis.

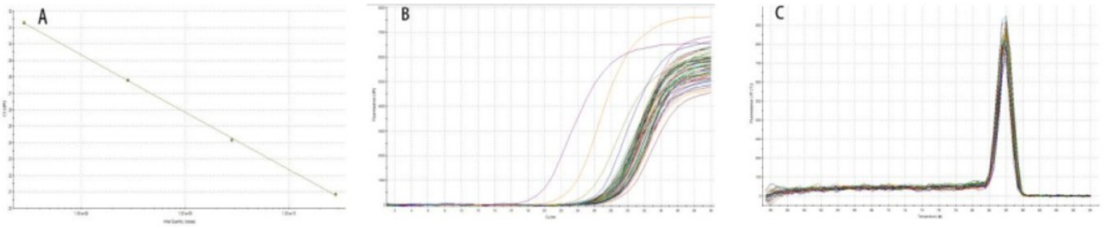

Figure S3. Analysis of the *Cry1Ac* gene by qRT-PCR. (A) Standard curve. (B) Amplification curve. (C) Melting curve.

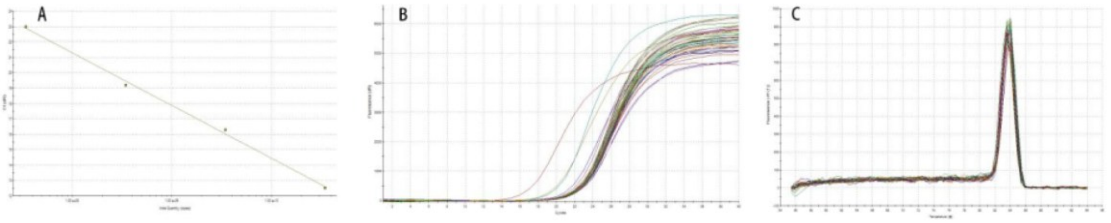

Figure S4. Analysis of the *Cry3A* gene by qRT-PCR. (A) Standard curve. (B) Amplification curve. (C) Melting curve.

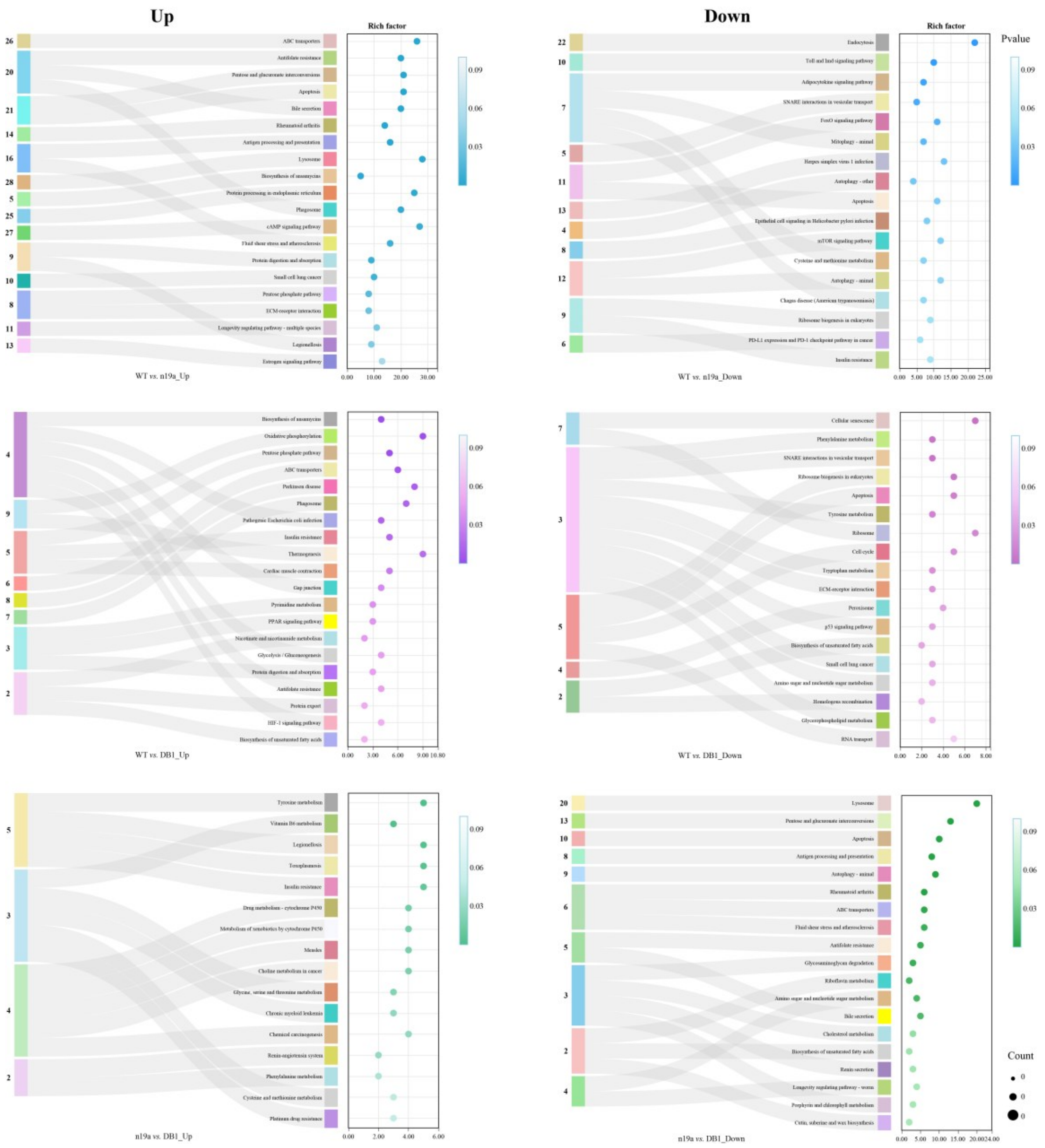

Figure S5. Significantly enriched KEGG pathways in each comparison group.
